# Cross-education does not accelerate the rehabilitation of neuromuscular functions after ACL reconstruction: a randomized controlled clinical trial

**DOI:** 10.1007/s00421-018-3892-1

**Published:** 2018-05-23

**Authors:** Tjerk Zult, Alli Gokeler, Jos J. A. M. van Raay, Reinoud W. Brouwer, Inge Zijdewind, Jonathan P. Farthing, Tibor Hortobágyi

**Affiliations:** 1 Center for Human Movement Sciences, University of Groningen, University Medical Center Groningen, Groningen, The Netherlands; 2 Vision and Eye Research Unit, School of Medicine, Anglia Ruskin University, Young Street 213, Cambridge, CB1 1PT UK; 3 Department of Orthopedic Surgery, Martini Hospital, Groningen, The Netherlands; 4 Department of Neuroscience, University of Groningen, University Medical Center Groningen, Groningen, The Netherlands; 5 College of Kinesiology, University of Saskatchewan, Saskatoon, Canada

**Keywords:** Force control, Maximal voluntary force, Postural stability, Proprioception, Strength training, Twitch interpolation

## Abstract

**Purpose:**

Cross-education reduces quadriceps weakness 8 weeks after anterior cruciate ligament (ACL) surgery, but the long-term effects are unknown. We investigated whether cross-education, as an adjuvant to the standard rehabilitation, would accelerate recovery of quadriceps strength and neuromuscular function up to 26 weeks post-surgery.

**Methods:**

Group allocation was randomized. The experimental (*n* = 22) and control (*n* = 21) group received standard rehabilitation. In addition, the experimental group strength trained the quadriceps of the non-injured leg in weeks 1–12 post-surgery (i.e., cross-education). Primary and secondary outcomes were measured in both legs 29 ± 23 days prior to surgery and at 5, 12, and 26 weeks post-surgery.

**Results:**

The primary outcome showed time and cross-education effects. Maximal quadriceps strength in the reconstructed leg decreased 35% and 12% at, respectively, 5 and 12 weeks post-surgery and improved 11% at 26 weeks post-surgery, where strength of the non-injured leg showed a gradual increase post-surgery up to 14% (all *p* ≤ 0.015). Limb symmetry deteriorated 9–10% more for the experimental than control group at 5 and 12 weeks post-surgery (both *p* ≤ 0.030). One of 34 secondary outcomes revealed a cross-education effect: Voluntary quadriceps activation of the reconstructed leg was 6% reduced for the experimental vs. control group at 12 weeks post-surgery (*p* = 0.023). Both legs improved force control (22–34%) and dynamic balance (6–7%) at 26 weeks post-surgery (all *p* ≤ 0.043). Knee joint proprioception and static balance remained unchanged.

**Conclusion:**

Standard rehabilitation improved maximal quadriceps strength, force control, and dynamic balance in both legs relative to pre-surgery but adding cross-education did not accelerate recovery following ACL reconstruction.

## Introduction

Anterior cruciate ligament (ACL) rupture is the most common knee injury, especially in adults aged 20–29 years with a preference for sports that involve pivoting, jumping, and direct contact between competitors (Majewski et al. 2006). Reconstruction of the ACL restores knee stability but deficits in knee extensor strength, neuromuscular control, and proprioception remain up to 2 years after surgery (Nagelli and Hewett 2017). These deficits are present also in the contralateral non-injured leg (Chung et al. 2015; Lepley et al. 2015; Negahban et al. 2014; Zult et al. 2017), suggesting that rehabilitation following ACL reconstruction should target both legs.

Bilateral impairments likely are the result of aberrations in the sensorimotor system following an ACL injury and reconstruction (Needle et al. 2017; Nyland et al. 2017). Maladaptive changes in somatosensory areas following ACL injury and reconstruction contribute to decreased knee joint proprioception (Baumeister et al. 2008; Valeriani et al. 1999), whereas a bilateral decrease in motor cortex excitability likely contributes to quadriceps weakness and activation failure (Lepley et al. 2015; Pietrosimone et al. 2015). Traditional ACL rehabilitation programs do not target these bilateral changes at the cortical and functional level, which can persist up to 48 months post-surgery (Lepley et al. 2015; Pietrosimone et al. 2015). Therefore, there is a need for intervention studies that aim to enhance quadriceps function by improving the descending drive from the motor cortex to the motoneuron pool of the quadriceps (Needle et al. 2017). Cross-education, which is the increase in muscle force on the untrained side after resistance training of the contralateral homologous limb muscle (Carroll et al. 2006), might as an adjuvant to standard therapy, improve muscle function after ACL reconstruction by increasing the neural drive to muscles of the reconstructed and non-injured leg (Hendy and Lamon 2017).

Cross-education in addition to standard care has been shown to improve rehabilitation outcomes in patients with different orthopaedic injuries (Magnus et al. 2013; Papandreou et al. 2009, 2013). To illustrate, ACL reconstructed patients had less quadriceps weakness 8 weeks after surgery (Papandreou et al. 2013), healthy subjects revealed attenuated strength loss and atrophy of the upper extremity muscles following 3 weeks of immobilization (Andrushko et al. 2017; Farthing et al. 2009), and wrist fracture patients exhibited increased strength and range of motion at 12 weeks post-fracture (Magnus et al. 2013). However, it is unknown whether cross-education of muscle force following an orthopaedic injury can improve voluntary muscle activation, postural stability, and force control—typical deficits present in the leg recovering from an ACL surgery (Nagelli and Hewett 2017; Telianidis et al. 2014).

Such deficits are all associated with altered muscle activation patterns and reduced lower extremity muscle strength (Clagg et al. 2015; Lepley et al. 2015; Pietrosimone et al. 2015; Telianidis et al. 2014) and can potentially be targeted by improving the motor commands of descending motor pathways (Needle et al. 2017). Strength training improved quadriceps force control at sub-maximal force levels in healthy old adults (Hortobagyi et al. 2001), whereas cross-education in healthy individuals increased quadriceps strength in the non-exercised leg with a trend toward greater quadriceps activation (Lepley and Palmieri-Smith 2014). However, targeting quadriceps weakness is difficult in the early phase after ACL reconstruction due to knee pain, effusion, and concerns about graft elongation when loading the quadriceps (van Melick et al. 2016). Therefore, cross-education training in the early phase of ACL rehabilitation could be of advantage in reducing quadriceps weakness. Especially, because cross-education increases muscle strength in the non-exercised muscles by increasing the neural drive to the contralateral non-exercised muscles and by inducing cortical adaptations in motor areas (Zult et al. 2014). Maladaptation in these motor networks is associated with poor neuromuscular function after ACL reconstruction (Needle et al. 2017) and cross-education training in the early phase of ACL rehabilitation could help to avoid this maladaptation.

We examined whether cross-education can accelerate the recovery of neuromuscular function when added to the standard care program in the early phase after ACL surgery. We expected that ACL patients subjected to additional strength training of the non-injured leg would show attenuated quadriceps strength loss (primary outcome) and less impaired voluntary quadriceps activation, quadriceps force control, and single-leg balance. Cross-education training would not improve knee joint proprioception as it is unlikely that sensory feedback mechanisms are involved in cross-education.

## Materials and methods

### Patients

Patients awaiting ACL reconstructive surgery were recruited during a 2-year period from the Martini Hospital in Groningen, The Netherlands. The patients who met the inclusion criteria were invited to take part in the study. Inclusion criteria were: age between 18 and 60 years, unilateral ACL tear with/without partial meniscal resection, time between ACL injury and testing < 2 years, autograft, allograft or artificial graft of any source, and weekly attendance of at least one supervised rehabilitation session. Patient exclusion criteria were: previous ACL reconstruction, history of a lower limb injury that required surgery, pregnancy, current or prior neurological conditions. The Tegner activity score was used to determine the physical activity level pre- and post-injury (Tegner and Lysholm 1985). The Waterloo Footedness questionnaire was used to determine leg dominance (Elias et al. 1998). In accordance with the Declaration of Helsinki, all patients provided written informed consent to the experimental procedures, which were approved by the medical ethics committee of the University Medical Center Groningen (ID 2012.362).

### Study design

This study was a randomized controlled clinical trial with measurements performed at 29 ± 23 days prior to surgery and at 5, 12, and 26 weeks post-surgery. Patients were randomly assigned to one of two parallel groups in a 1:1 ratio, to receive either the standard care or standard care plus cross-education intervention. An investigator not involved in data collection generated a randomization list on the computer which was then used by an independent physiotherapist for group allocation. Group allocation was performed after surgery and before patients commenced rehabilitation. Orthopaedic surgeons and data collectors were blinded to patients’ group assignment. This randomized clinical trial is registered at the Dutch trial register (http://www.trialregister.nl) under NTR4395.

### Intervention

The experimental and control group performed the rehabilitation protocol at the same outpatient physical therapy clinic. Both groups received standardized rehabilitation. In the first 4 weeks after ACL reconstruction, the protocol aimed to reduce inflammation and swelling, restore full knee extension, and facilitate quadriceps activity. In weeks 4–12, the goals were to strengthen the quadriceps and hamstring muscles using resistance training and to improve balance and core stability. In 12–24 weeks, rehabilitation continued with more advanced balance and core stability exercises, resistance training with a focus on hypertrophy, running with minimal directional change, and two-legged jumping tasks. In weeks 24–36, the program incorporated running with agility drills, single-leg jumps, and power training focused on reducing strength deficits. In addition, the experimental group performed the leg press and leg extension exercise with the non-injured leg on standard gym machines with the focus on the concentric part of the exercise (i.e., cross-education training). Both exercises consisted of three sets at an 8–12 repetition maximum with 1–2 min rest between sets. These two cross-education exercises were performed in every training session of weeks 1–12 after ACL surgery to maximize hypertrophy of the quadriceps muscles (American College of Sports Medicine 2009). The total number of repetitions varied between patients and was not reported. There was a gradual build up in resistance to ensure that the patients received an adequate training stimulus. The patients trained twice a week under supervision of a physiotherapist.

### Outcome measures

#### Primary outcome

Isometric quadriceps maximal voluntary contractions (MVCs) were measured in each leg at 65° on an isokinetic dynamometer (Biodex Medical Systems, Shirley, NY, USA) using an established protocol (Zult et al. 2017). The strength testing started after 5 min of warm-up on a bicycle ergometer. Strength testing always preceded the assessment of the secondary outcomes. Patients performed two familiarization trials at 50% of their estimated MVC followed by three maximal quadriceps contractions. Patients had a 1-min break between repetitions. The starting leg was randomly chosen and this randomization was carried forward to the secondary outcomes and subsequent test sessions. Statistical analysis was performed on the peak torque normalized to body weight. Test–retest reliability of these measurements is good to excellent (Hortobagyi et al. 2001, 2004). The percentage of MVC change was also analysed for each leg and between legs (i.e., limb symmetry index). The limb symmetry index was calculated as: (reconstructed leg/non-injured leg) * 100%.

#### Secondary outcomes

The secondary outcomes were voluntary quadriceps activation, quadriceps force accuracy and variability, knee joint proprioception, and single-leg balance (see details below). All secondary outcomes were examined in each leg in one of three random orders. The randomization was carried forward to subsequent testing sessions.

#### Voluntary quadriceps activation

Quadriceps activation was examined using the twitch interpolation technique and the central activation ratio (CAR) as detailed previously (Behm et al. 1996, 2001; Zult et al. 2017). Eleven patients experienced the electrical stimulation as unpleasant, and therefore, only a subsample of patients could be tested (experimental: *n* = 17; control: *n* = 15). Patients were seated on a custom-built dynamometer (Verkerke et al. 2003) with the hips and knees in 90° flexion. The quadriceps was stimulated using two 10 × 14 cm aluminium foil electrodes covered with water-soaked sponges (cathode: middle of rectus femoris, anode: distal 10 cm above patella). Single rectangular pulses of 200 µs duration and 10–1000 mA amplitude were delivered via the electrodes by a high-voltage stimulator (Digitimer DS7AH, Welwyn Garden City, UK). Doublets were elicited with a 10 ms interval between the two pulses. The force evoked by a doublet is referred to as twitch. Quadriceps torques were amplified and sampled at 500 Hz (CED Power 1401 Plus; Cambridge Electronic Design, Cambridge, UK), monitored on a computer screen, and recorded and analysed using accompanying software (Spike 2, version 5.21). The protocol comprised: (1) three isometric MVCs; (2) determination of the maximal twitch torque during contractions at 10% MVC (to eliminate slack); (3) superimposed twitches at 30, 50, 75, and 100% of MVC; (4) two twitches at rest from which the higher of the two was classified as potentiated twitch.

To generate a linear regression equation for each patient, the twitch expressed as a percentage of the potentiated twitch was plotted against the respective force upon which the twitch was superimposed. The intersection point with the *x*-axis is classified as the estimated MVC (Behm et al. 2001). The CAR was calculated as: MVC/(MVC + superimposed twitch) * 100%.

#### Quadriceps force control

A target-matching task, with acceptable test–retest reliability (Hortobagyi et al. 2001, 2004), was performed with the target set to 20% MVC for isometric trials and to 40 Nm for dynamic trials (Zult et al. 2017). After familiarization, patients performed three isometric trials at 65° of knee flexion (5-s duration) and four concentric and eccentric trials at 20°/s between 10° and 90° of knee flexion. Force accuracy and force variability were determined over the final 3-s portion of the data for isometric trials and over the middle 2-s portion for concentric and eccentric trials. Force accuracy was the absolute difference between the produced torque and the target torque. Force variability was the coefficient of variation [i.e., standard deviation (SD) of the produced force divided by the mean force]. The mean across the trials was used in the statistical analysis.

#### Knee joint proprioception

We measured proprioception with a joint repositioning task at four randomized target positions (15°, 30°, 45°, and 60° of knee flexion) (Hortobagyi et al. 2004). Every target position was tested once. Knee joint proprioception was calculated as the absolute difference between the actual joint angle and the target position and was expressed in degrees. Test–retest reliability is acceptable (Hortobagyi et al. 2004).

#### Single-leg balance

Static balance was tested with the one-leg standing balance test, starting with the eyes open followed by the eyes-closed condition (Atwater et al. 1990). Patients had two attempts per condition with a 1-min rest period between trials. The maximum score that could be obtained was 60 s. The best score per condition was used in the statistical analysis. This test has acceptable test–retest reliability (Atwater et al. 1990).

Dynamic balance was examined with the star-excursion balance test (Gribble and Hertel 2003). The Star-excursion balance test was performed in clockwise direction and the starting line was randomly determined. Each leg was tested three times. After test completion of one leg, there was a 5-min break before the other leg was tested. The mean reaching distance was computed across the three trials and normalized to leg length. The composite score, which is the average score across the eight lines, was used in the statistical analysis (Zult et al. 2017). Test–retest reliability for the star-excursion balance test differed per direction from acceptable to excellent (Hertel et al. 2000).

### Data analysis

We performed an a priory power analysis with G*Power 3.1 to calculate the required sample size necessary to obtain a significant group by time effect on the primary outcome measure (i.e., maximal quadriceps torque). The effect of cross-education on quadriceps MVCs has only been examined in highly trained soldiers and not in recreational athletes. Therefore, a small effect size of 0.2 was used for the power analysis to prevent underestimation of the sample size. The calculated sample size was 36 (i.e., 18 patients per group) based on an effect size of 0.2 with a power of 80% at the *p* < 0.05 significance level. We aimed for 25 patients per group to allow for dropouts.

Data in the text and figures are presented as mean ± SD. A modified intention-to-treat analysis was executed including all patients who were randomized for treatment and attended minimal two test sessions. Normality was checked for each variable. The analyses were executed on log-transformed data for force accuracy and variability, because normality was violated. Differences in group characteristics were examined with a one-way ANOVA when measured on a ratio scale and with a Kruskall–Wallis or Chi square test when measured on, respectively, an ordinal or nominal scale.

The primary and secondary outcomes were analysed using multilevel analysis (SPSS version 23), because 8% of the data points were missing. Multilevel analysis can deal with incomplete data sets in contrast to repeated measures analysis of variance (Rasbash et al. 2009) and handles baseline differences between groups by allowing intercepts to vary between patients. A random intercept and slope model was used, where repeated measurements (level 1) were nested within individual ACL patients (level 2). Subsequently, the following explanatory variables were added to the model: group (experimental group, control group [as reference]), time (pre-surgery [as reference], 5, 12, and 26 weeks post-surgery), and the group by time interaction. Separate analyses were performed for the reconstructed leg and non-injured leg. Gender was added as covariate for quadriceps MVCs and voluntary quadriceps activation. The maximum likelihood method was used to estimate the parameters of the multilevel model. Explanatory variables that significantly contributed to the model were subjected to a Bonferroni post hoc test to determine the means that were different. Cohen’s *d* and 95% confidence intervals (CI) were calculated for significant effects. The level of significance (*α*) was set at *p* < 0.05.

## Results

### Patients

**Fig. 1:**
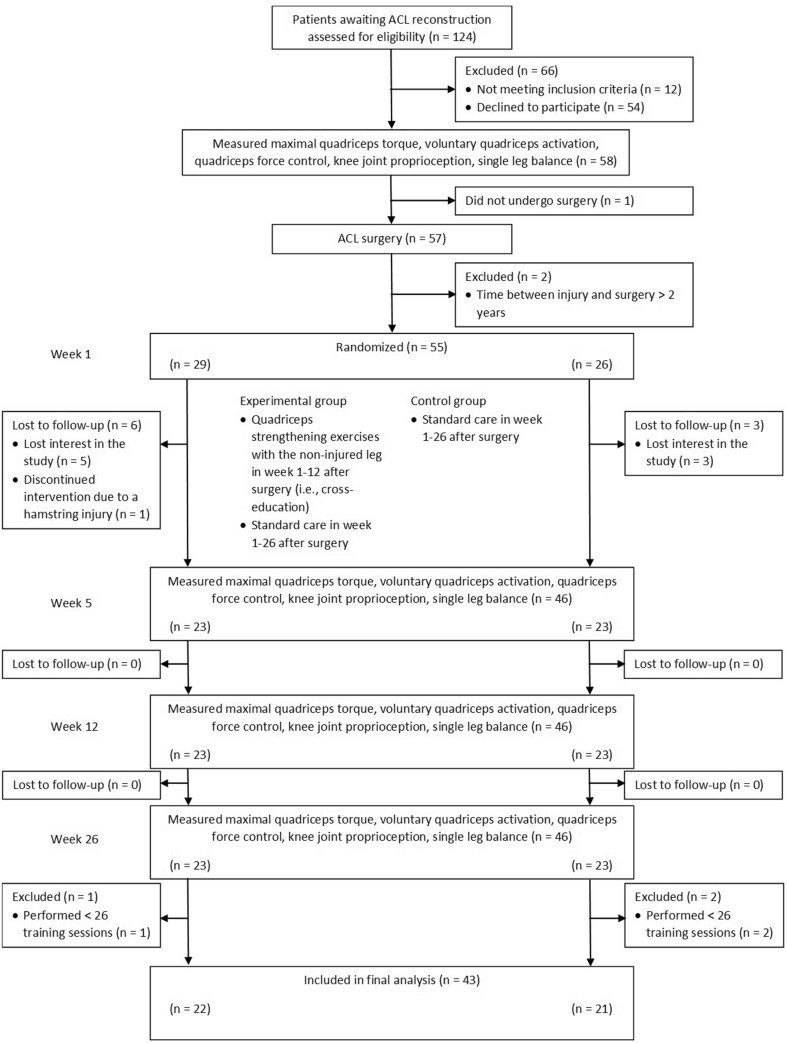
Flow diagram of patient enrolment

Figure 1 shows the flow of patient enrolment (*n* = 55 enrolled) and Table 1 shows the group characteristics. Four patients deviated from the treatment protocol by receiving the control group treatment while allocated to the experimental group.

**Table 1:** Mean (SD) group characteristics of the participants

	Experimental group (*n* = 22)	Control group (*n* = 21)	*p* value
Age (years)	28 (9)	28 (10)	0.896
Sex (male/female)	16/6	8/14	0.022*
Mass (kg)	82 (13)	74 (10)	0.029*
Height (cm)	182 (8)	175 (6)	0.002*
BMI (kg/m^2^)	25 (3)	24 (3)	0.635
Leg dominance (left/right)	3/19	3/18	0.951
Operated leg (left/right)	9/13	7/14	0.607
Graft type (hamstring tendon/bone-patellar tendon bone/artificial)	18/3/1	19/2/0	0.548
Tegner score pre-injury	8 (2)	7 (2)	0.275
Tegner score post-injury	4 (1)	4 (1)	0.533
Number of training sessions	44 (11)	50 (12)	0.073
Time between injury and testing (days)	189 (138)	160 (95)	0.440
Time between testing and surgery (days)	28 (28)	30 (17)	0.773

*Group difference (*p* < 0.05)

### Primary outcome

**Fig. 2:**
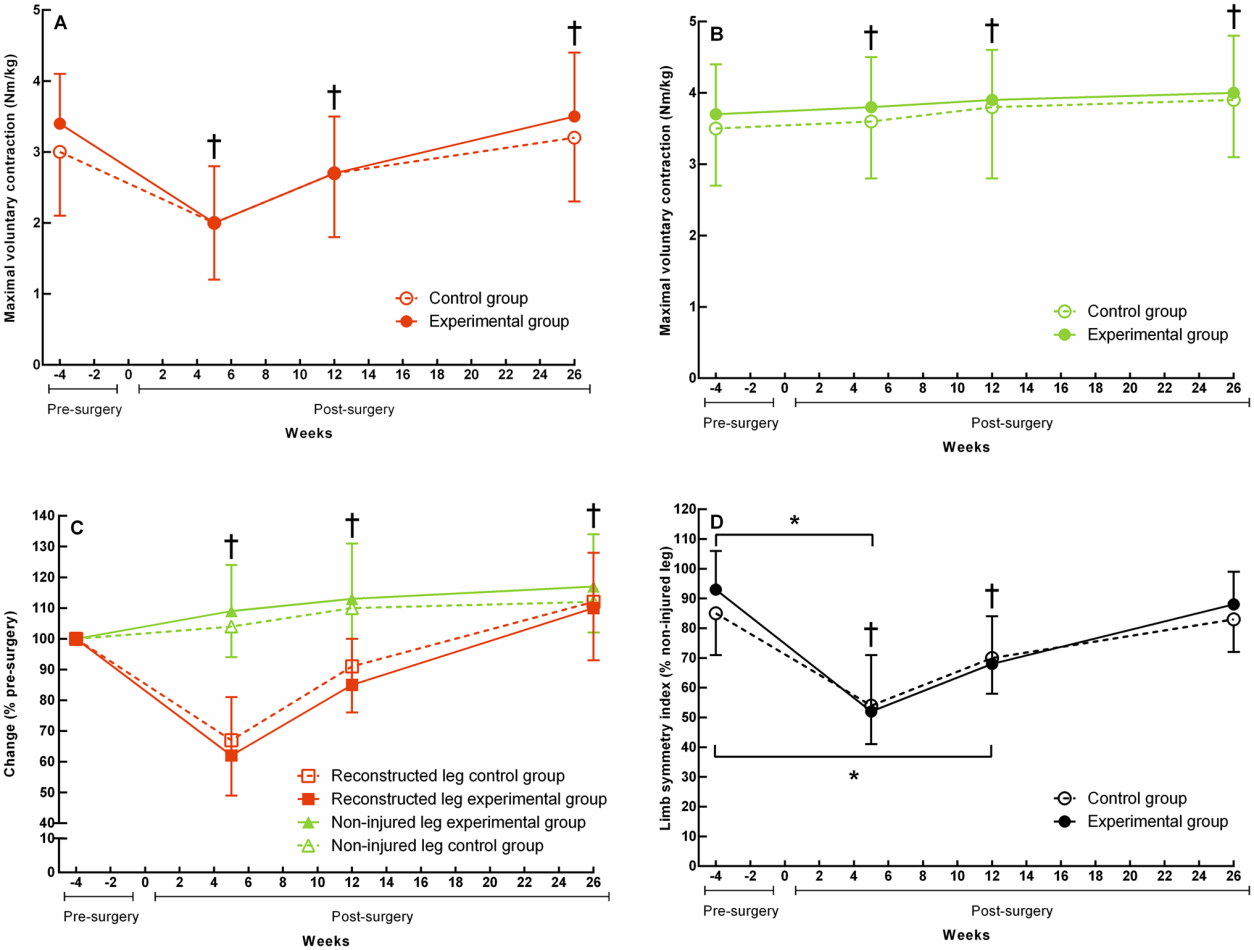
Isometric quadriceps MVC means (SD) of the experimental group (filled symbols) and control group (open symbols). The colour of the lines and symbols represent whether the values are for the reconstructed leg (red), non-injured leg (green), or limb symmetry index (black). **a** Quadriceps MVCs of the reconstructed leg. **b** Quadriceps MVCs of the non-injured leg. **c** Percentage change scores of the reconstructed an non-injured leg. **d** Limb symmetry indices for quadriceps MVCs. *Group by time interaction (*p* < 0.05); ^†^different compared to pre-surgery (*p* < 0.05). (Color figure online)

Figure 2a shows the time main effects for the quadriceps MVCs of the reconstructed leg (*F*_3,117_ = 107.0, *p* < 0.001). Post hoc testing revealed that quadriceps MVCs, relative to pre-surgery, were decreased at 5 weeks post-surgery (95% CI [− 1.4, − 0.9], *d* = − 1.50) and at 12 weeks post-surgery (95% CI [− 0.7, − 0.2], *d* = − 0.59) and improved at 26 weeks post-surgery (95% CI [0.0, 0.5], *d* = 0.23) (all *p* ≤ 0.015). Figure 2b illustrates the time main effects for quadriceps MVCs of the non-injured leg (*F*_3,119_ = 28.5, *p* < 0.001). Compared to pre-surgery, MVCs increased at all time points post-surgery (all *p* ≤ 0.001, *d* range = [0.13, 0.40]). Figure 2c shows the time effects for the percentage change scores in the reconstructed and non-injured leg (both *p* < 0.001). Quadriceps MVCs in the reconstructed leg, relative to pre-surgery, were 35% decreased at 5 weeks post-surgery, 12% decreased at 12 weeks post-surgery and 11% improved at 26 weeks post-surgery (all *p* ≤ 0.002). Quadriceps MVCs in the non-injured leg, relative to pre-surgery, were 6% improved at 5 weeks post-surgery, 12% improved at 12 weeks post-surgery, and 14% improved at 26 weeks post-surgery (all *p* ≤ 0.002). Figure 2d reveals the borderline significant group by time interaction for the limb symmetry index (*F*_3,118_ = 2.5, *p* = 0.060). Relative to pre-surgery, the decrease in limb symmetry was 10% more for the experimental vs. control group at 5 weeks post-surgery (95% CI 2–18, *d* = − 0.77, *p* = 0.017) and 9% more for the experimental than control group at 12 weeks post-surgery (95% CI 1–17, *d* = − 0.69, *p* = 0.030). The time effect for the limb symmetry index shows that limb symmetry, compared to pre-surgery, was 36% decreased at 5 weeks post-surgery (*p* < 0.001), 19% decreased at 12 weeks post-surgery (*p* < 0.001), and returned to pre-surgery level at 26 weeks post-surgery (*p* = n.s.).

### Secondary outcomes

#### Voluntary quadriceps activation

Table 2 illustrates the voluntary quadriceps activation in the ACL patients’ reconstructed and non-injured leg. A between-group difference was observed for the CAR of the reconstructed leg (*F*_3,85_ = 4.7, *p* = 0.004). Post hoc testing showed that the CAR decreased 6% in the experimental group from pre-surgery to 12 weeks post-surgery, while the control group revealed no change (95% CI [− 12, − 1], *d* = − 0.66, *p* = 0.023). Main effects of time were observed for the reconstructed leg only (all *p* < 0.001). Post hoc testing revealed impairments 5 and 12 weeks post-surgery relative to pre-surgery (all *p* ≤ 0.019).

**Table 2:** Quadriceps voluntary force and muscle activation

Outcome	Groups							
	Pre-surgery		Week 5 post-surgery		Week 12 post-surgery		Week 26 post-surgery	
	Exp (*n* = 17)	Con (*n* = 15)	Exp (*n* = 17)	Con (*n* = 15)	Exp (*n* = 17)	Con (*n* = 15)	Exp (*n* = 17)	Con (*n* = 15)
Central activation ratio								
Reconstructed leg (%)	96 (2)	96 (3)	96 (3)	90 (10)	90 (12)	97 (3)	94 (7)	97 (3)
Non-injured leg (%)	97 (2)	97 (3)	97 (2)	97 (2)	97 (4)	97 (3)	96 (4)	98 (2)
Potentiated doublet force								
Reconstructed leg (Nm)	83 (29)	68 (20)	61 (21)	55 (20)	71 (22)	66 (19)	81 (29)	72 (23)
Non-injured leg (Nm)	90 (27)	78 (21)	92 (30)	74 (20)	89 (28)	78 (23)	90 (33)	78 (23)
Isometric MVC								
Reconstructed leg (Nm)	211 (86)	175 (65)	127 (63)	98 (68)	153 (72)	152 (62)	198 (97)	162 (63)
Non-injured leg (Nm)	227 (72)	192 (59)	219 (79)	186 (58)	235 (89)	202 (54)	234 (93)	196 (58)
Estimated MVC								
Reconstructed leg (Nm)	160 (64)	133 (43)	106 (50)	83 (45)	129 (44)	119 (41)	157 (68)	128 (47)
Non-injured leg (Nm)	179 (59)	149 (39)	169 (61)	140 (40)	181 (65)	156 (42)	182 (71)	153 (43)

Outcome	Difference within groups						Difference between groups		
	Week 5 minus pre-surgery		Week 12 minus pre-surgery		Week 26 minus pre-surgery		Week 5 minus pre-surgery	Week 12 minus pre-surgery	Week 26 minus pre-surgery
	Exp	Con	Exp	Con	Exp	Con	Exp-Con	Exp-Con	Exp-Con
Central activation ratio									
Reconstructed leg (%)	− 2 (9)	− 6 (7)^†^	− 6 (8)^†^	0 (7)	− 3 (8)	1 (7)	4 (− 2 to 10)	− 6 (− 12 to − 1)*	− 3 (− 9 to 2)
Non-injured leg (%)	0 (3)	0 (2)	− 1 (3)	0 (2)	− 1 (3)	1 (2)	− 1 (− 3 to 1)	− 1 (− 3 to 1)	− 2 (− 4 to 0)
Potentiated doublet force									
Reconstructed leg (Nm)	− 19 (19)^†^	− 14 (12)^†^	− 12 (17)	− 4 (12)	1 (18)	4 (12)	− 6 (− 16 to 5)	− 8 (− 18 to 3)	− 3 (− 13 to 7)
Non-injured leg (Nm)	2 (13)	− 2 (9)	− 2 (14)	0 (9)	3 (14)	2 (9)	4 (− 4 to 12)	− 2 (− 10 to 6)	1 (− 8 to 9)
Isometric MVC									
Reconstructed leg (Nm)	− 88 (57)	− 76 (40)^†^	− 62 (53)	− 28 (40)	− 12 (54)	− 11 (39)	− 12 (− 46 to 22)	− 33 (− 66 to − 1)	− 1 (− 34 to 32)
Non-injured leg (Nm)	− 8 (41)	− 5 (26)	3 (42)	7 (27)	11 (43)	5 (26)	− 3 (− 27 to 21)	− 4 (− 28 to 21)	5 (− 19 to 30)
Estimated MVC									
Reconstructed leg (Nm)	− 48 (39)^†^	− 48 (24)^†^	− 33 (36)	− 18 (24)^†^	2 (37)	− 3 (23)	0 (− 23 to 22)	− 15 (− 37 to 6)	5 (− 16 to 26)
Non-injured leg (Nm)	− 9 (30)	− 7 (16)	0 (31)	5 (16)	7 (31)	6 (16)	− 2 (− 19 to 15)	− 5 (− 23 to 12)	− 1 (− 16 to 18)

Mean (SD) of each group, mean (SD) difference within each group, and mean (95% CI) difference between groups

*Exp* experimental group, *Con* control group, *MVC* maximal voluntary contraction

*Group difference (*p* < 0.05), ^†^different compared to pre-surgery (*p* < 0.05)

#### Quadriceps force control

Table 3 demonstrates the quadriceps force accuracy and Table 4 demonstrates the quadriceps force variability in the ACL reconstructed and non-injured leg. Time main effects were observed (all *p* ≤ 0.039). Force accuracy and variability in both legs improved 13–56% over time relative to pre-surgery (*d* range [0.24, 0.90], all *p* ≤ 0.024), except force variability of the reconstructed leg which deteriorated 27% at 5 weeks post-surgery when measured during eccentric muscle contractions (*d* = − 0.39, *p* = 0.002).

**Table 3:** Quadriceps force accuracy

Outcome	Groups							
	Pre-surgery		Week 5 post-surgery		Week 12 post-surgery		Week 26 post-surgery	
	Exp (*n* = 22)	Con (*n* = 21)	Exp (*n* = 22)	Con (*n* = 21)	Exp (*n* = 22)	Con (*n* = 21)	Exp (*n* = 22)	Con (*n* = 21)
Eccentric 60°/s								
Reconstructed leg (Nm)	13 (8)	14 (6)	11 (5)	12 (6)	10 (6)	9 (4)	9 (5)	10 (6)
Non-injured leg (Nm)	12 (4)	13 (6)	10 (5)	11 (5)	8 (3)	9 (5)	8 (5)	8 (5)
Isometric								
Reconstructed leg (Nm)	2 (1)	3 (5)	1 (1)	1 (1)	1 (0)	1 (1)	1 (1)	2 (2)
Non-injured leg (Nm)	2 (1)	3 (5)	3 (4)	3 (4)	2 (1)	2 (1)	2 (1)	2 (1)
Concentric 60°/s								
Reconstructed leg (Nm)	7 (3)	15 (15)	7 (3)	7 (4)	7 (6)	7 (4)	6 (6)	9 (9)
Non-injured leg (Nm)	8 (5)	15 (12)	6 (4)	12 (10)	7 (5)	10 (11)	8 (4)	12 (12)

Outcome	Difference within groups						Difference between groups		
	Week 5 minus pre-surgery		Week 12 minus pre-surgery		Week 26 minus pre-surgery		Week 5 minus pre-surgery	Week 12 minus pre-surgery	Week 26 minus pre-surgery
	Exp	Con	Exp	Con	Exp	Con	Exp-Con	Exp-Con	Exp-Con
Eccentric 60°/s									
Reconstructed leg (Nm)	− 1 (5)	− 1 (6)	− 3 (5)^†^	− 5 (6)^†^	− 5 (5)^†^	− 4 (5)^†^	0 (− 3 to 3)	1 (− 2 to 4)	− 1 (− 4 to 2)
Non-injured leg (Nm)	− 2 (4)^†^	− 2 (5)	− 5 (4)^†^	− 4 (5)^†^	− 5 (4)^†^	− 5 (5)^†^	0 (− 2 to 3)	− 1 (− 3 to 2)	0 (− 2 to 3)
Isometric									
Reconstructed leg (Nm)	− 1 (1)^†^	− 2 (4)^†^	− 1 (1)^†^	− 2 (4)^†^	0 (1)	− 1 (4)	1 (0 to 3)	1 (0 to 3)	1 (− 1 to 3)
Non-injured leg (Nm)	1 (3)	− 1 (4)	0 (3)	− 2 (4)	0 (3)	− 2 (4)	1 (− 1 to 3)	1 (− 1 to 3)	2 (0 to 4)
Concentric 60°/s									
Reconstructed leg (Nm)	0 (6)	− 8 (12)^†^	0 (6)	− 8 (12)^†^	− 1 (6)	− 7 (11)^†^	8 (− 2 to 14)	8 (− 2 to 14)	6 (0 to 12)
Non-injured leg (Nm)	− 1 (5)	− 3 (8)	− 2 (5)^†^	− 5 (9)^†^	− 2 (5)	− 3 (8)	3 (− 2 to 7)	2 (− 2 to 7)	1 (− 3 to 6)

Mean (SD) of each group, mean (SD) difference within each group, and mean (95% CI) difference between groups

Force accuracy is expressed as the absolute difference between the produced force and the target force

*Exp* experimental group, *Con* control group

^†^Different compared to pre-surgery (*p* < 0.05)

**Table 4:** Quadriceps force variability

Outcome	Groups							
	Pre-surgery		Week 5 post-surgery		Week 12 post-surgery		Week 26 post-surgery	
	Exp (*n* = 22)	Con (*n* = 21)	Exp (*n* = 22)	Con (*n* = 21)	Exp (*n* = 22)	Con (*n* = 21)	Exp (*n* = 22)	Con (*n* = 21)
Eccentric 60°/s								
Reconstructed leg (Nm)	21 (12)	27 (19)	31 (12)	31 (15)	23 (17)	22 (13)	18 (8)	17 (8)
Non-injured leg (Nm)	21 (7)	22 (13)	17 (10)	21 (14)	15 (5)	19 (10)	16 (6)	15 (6)
Isometric								
Reconstructed leg (Nm)	3 (1)	3 (1)	3 (1)	3 (1)	2 (1)	3 (1)	2 (1)	2 (1)
Non-injured leg (Nm)	3 (1)	3 (1)	3 (1)	3 (1)	2 (1)	3 (1)	3 (3)	3 (1)
Concentric 60°/s								
Reconstructed leg (Nm)	16 (8)	18 (7)	14 (7)	15 (9)	13 (7)	14 (8)	13 (7)	13 (5)
Non-injured leg (Nm)	21 (9)	17 (8)	16 (9)	14 (6)	10 (4)	13 (9)	11 (6)	14 (6)

Outcome	Difference within groups						Difference between groups		
	Week 5 minus pre-surgery		Week 12 minus pre-surgery		Week 26 minus pre-surgery		Week 5 minus pre-surgery	Week 12 minus pre-surgery	Week 26 minus pre-surgery
	Exp	Con	Exp	Con	Exp	Con	Exp-Con	Exp-Con	Exp-Con
Eccentric 60°/s									
Reconstructed leg (Nm)	8 (12)^†^	5 (16)	0 (11)	− 5 (16)	− 6 (12)	− 10 (15)^†^	4 (− 5 to 12)	5 (− 4 to 14)	5 (− 4 to 13)
Non-injured leg (Nm)	− 4 (9)^†^	− 2 (11)	− 6 (9)^†^	-4 (12)	− 5 (9)^†^	− 7 (12)^†^	− 2 (− 8 to 4)	− 2 (− 8 to 4)	2 (− 4 to 9)
Isometric									
Reconstructed leg (Nm)	0 (1)	0 (1)	− 1 (1)^†^	− 1 (1)^†^	− 1 (1)^†^	− 1 (1)^†^	0 (0 to 1)	0 (− 1 to 1)	0 (− 1 to 1)
Non-injured leg (Nm)	0 (2)	0 (1)	− 1 (2)	0 (1)	0 (2)	− 1 (1)^†^	0 (− 1 to 1)	0 (− 1 to 1)	1 (0 to 2)
Concentric 60°/s									
Reconstructed leg (Nm)	− 1 (8)	− 3 (10)	− 3 (8)	− 4 (10)	− 3 (9)	− 5 (10)	2 (− 4 to 7)	1 (− 4 to 7)	2 (− 3 to 8)
Non-injured leg (Nm)	− 5 (9)	− 2 (10)	− 11 (9)^†^	− 4 (10)	− 10 (9)^†^	− 3 (10)	− 2 (− 8 to 3)	− 7 (− 12 to 1)	− 7 (− 12 to − 1)

Mean (SD) of each group, mean (SD) difference within each group, and mean (95% CI) difference between groups

Force variability was quantified by the SD of the produced force divided by the mean force (i.e., coefficient of variation)

*Exp* experimental group, *Con* control group

^†^Different compared to pre-surgery (*p* < 0.05)

#### Knee joint proprioception

Table 5 shows the knee joint proprioception of the ACL patients’ reconstructed and non-injured leg. A time main effect was observed for the non-injured leg at a target angle of 60° (*F*_3,123_ = 4.3, *p* = 0.006); knee joint proprioception was better at 5 weeks post-surgery than pre-surgery (*d* = 0.39, *p* = 0.018).

**Table 5:** Knee joint proprioception

Outcome	Groups							
	Pre-surgery		Week 5 post-surgery		Week 12 post-surgery		Week 26 post-surgery	
	Exp (*n* = 22)	Con (*n* = 21)	Exp (*n* = 22)	Con (*n* = 21)	Exp (*n* = 22)	Con (*n* = 21)	Exp (*n* = 22)	Con (*n* = 21)
15°								
Reconstructed leg (°)	3 (3)	3 (3)	3 (2)	3 (3)	3 (2)	3 (4)	3 (3)	3 (2)
Non-injured leg (°)	4 (3)	3 (2)	3 (2)	4 (3)	3 (3)	3 (3)	3 (3)	3 (3)
30°								
Reconstructed leg (°)	2 (2)	4 (3)	3 (2)	3 (3)	4 (3)	4 (3)	5 (2)	3 (3)
Non-injured leg (°)	4 (3)	3 (2)	4 (2)	4 (2)	3 (3)	3 (3)	2 (2)	2 (2)
45°								
Reconstructed leg (°)	5 (4)	4 (3)	4 (2)	3 (2)	3 (2)	3 (2)	2 (3)	3 (4)
Non-injured leg (°)	3 (2)	3 (3)	4 (4)	4 (3)	2 (2)	4 (3)	2 (2)	3 (3)
60°								
Reconstructed leg (°)	2 (2)	2 (2)	3 (2)	3 (2)	3 (3)	3 (3)	3 (3)	3 (2)
Non-injured leg (°)	4 (3)	3 (2)	2 (2)	2 (2)	3 (2)	3 (2)	4 (3)	4 (3)

Outcome	Difference within groups						Difference between groups		
	Week 5 minus pre-surgery		Week 12 minus pre-surgery		Week 26 minus pre-surgery		Week 5 minus pre-surgery	Week 12 minus pre-surgery	Week 26 minus pre-surgery
	Exp	Con	Exp	Con	Exp	Con	Exp-Con	Exp-Con	Exp-Con
15°									
Reconstructed leg (°)	0 (3)	0 (4)	0 (3)	0 (4)	0 (3)	− 1 (4)	0 (− 2 to 2)	0 (− 3 to 2)	1 (− 1 to 3)
Non-injured leg (°)	− 1 (4)	1 (3)	− 1 (4)	0 (4)	− 1 (4)	0 (3)	− 2 (-4 to 1)	− 1 (− 3 to 1)	− 1 (− 3 to 1)
30°									
Reconstructed leg (°)	1 (3)	− 1 (4)	2 (3)	0 (4)	2 (3)^†^	0 (4)	1 (− 1 to 3)	1 (− 1 to 3)	3 (1 to 5)
Non-injured leg (°)	0 (3)	0 (4)	− 1 (3)	2 (4)	− 1 (4)	1 (4)	0 (− 2 to 2)	− 2 (− 5 to 0)	− 2 (− 5 to 0)
45°									
Reconstructed leg (°)	− 1 (4)	0 (4)	− 1 (4)	0 (4)	− 2 (4)	− 1 (4)	0 (− 3 to 2)	− 1 (− 4 to 1)	− 1 (− 4 to 1)
Non-injured leg (°)	0 (4)	1 (3)	− 1 (4)	1 (3)	− 1 (4)	0 (3)	0 (− 2 to 2)	− 2 (− 4 to 0)	− 1 (− 3 to 1)
60°									
Reconstructed leg (°)	0 (3)	0 (3)	1 (3)	1 (3)	0 (3)	1 (3)	0 (− 2 to 2)	0 (− 2 to 2)	0 (− 2 to 2)
Non-injured leg (°)	− 2 (3)^†^	− 1 (3)	− 1 (3)	0 (3)	0 (4)	1 (3)	− 1 (− 3 to 1)	− 1 (− 3 to 1)	− 1 (− 3 to 1)

Mean (SD) of each group, mean (SD) difference within each group, and mean (95% CI) difference between groups

*Exp* experimental group, *Con* control group

^†^Different compared to pre-surgery (*p* < 0.05)

#### Single-leg balance

Table 6 shows the single-leg balance of the ACL patients’ reconstructed and non-injured leg. No significant effects were observed for one-leg standing balance in the eyes open and eyes-closed condition (all *p* ≥ 0.137). The star-excursion balance test revealed a time main effect in both legs (all *p* < 0.001). The composite score of the reconstructed leg, relative to pre-surgery, showed 4% deficit 5 weeks post-surgery (95% CI [− 7, − 2], *d* = − 0.53) and 5% improvement 26 weeks post-surgery (95% CI [3, 8], *d* = 0.58). The composite score of the non-injured leg, relative to pre-surgery, increased 3% at 12 weeks post-surgery (95% CI [1, 6], *d* = 0.5) and 5% at 26 weeks post-surgery (95% CI [3, 8], *d* = 0.75) (all *p* ≤ 0.001).

**Table 6:** Single-leg balance

Outcome	Groups							
	Pre-surgery		Week 5 post-surgery		Week 12 post-surgery		Week 26 post-surgery	
	Exp (*n* = 22)	Con (*n* = 21)	Exp (*n* = 22)	Con (*n* = 21)	Exp (*n* = 22)	Con (*n* = 21)	Exp (*n* = 22)	Con (*n* = 21)
One-leg standing balance, eyes open (s)								
Reconstructed leg	60 (0)	60 (0)	59 (4)	60 (1)	60 (0)	60 (0)	60 (0)	60 (0)
Non-injured leg	60 (0)	60 (0)	60 (0)	60 (0)	60 (0)	60 (0)	60 (2)	60 (0)
One-leg standing balance, eyes closed (s)								
Reconstructed leg	22 (18)	31 (21)	26 (22)	28 (20)	25 (21)	32 (21)	23 (21)	33 (19)
Non-injured leg	25 (22)	34 (21)	28 (24)	33 (20)	30 (22)	35 (20)	32 (22)	34 (20)
Star-excursion balance test, composite score (% leg length)^a^								
Reconstructed leg	80 (8)	79 (6)	75 (7)	77 (9)	82 (8)	83 (9)	84 (12)	86 (7)
Non-injured leg	82 (9)	80 (8)	82 (9)	83 (9)	85 (8)	85 (8)	86 (9)	88 (7)

	Difference within groups						Difference between groups		
	Week 5 minus pre-surgery		Week 12 minus pre-surgery		Week 26 minus pre-surgery		Week 5 minus pre-surgery	Week 12 minus pre-surgery	Week 26 minus pre-surgery
	Exp	Con	Exp	Con	Exp	Con	Exp-Con	Exp-Con	Exp-Con
One-leg standing balance, eyes open (s)									
Reconstructed leg	− 1 (3)	0 (1)	0 (3)	0 (1)	0 (3)	0 (1)	− 1 (− 2 to 1)	0 (− 1 to 1)	0 (− 1 to 1)
Non-injured leg	0 (1)	0 (1)	0 (1)	0 (1)	− 1 (1)	0 (1)	0 (− 1 to 1)	0 (− 1 to 1)	− 1 (− 1 to 0)
One-leg standing balance, eyes closed (s)									
Reconstructed leg	4 (20)	− 3 (19)	5 (19)	2 (19)	3 (21)	2 (19)	7 (− 4 to 19)	3 (− 8 to 15)	0 (− 12 to 12)
Non-injured leg	3 (15)	− 2 (19)	6 (15)	3 (19)	10 (16)^†^	0 (19)	5 (− 5 to 15)	3 (− 7 to 13)	9 (− 2 to 20)
Star-excursion balance test, composite score (% leg length)^a^									
Reconstructed leg	− 5 (6)^†^	− 3 (6)	1 (6)	4 (6)	4 (6)^†^	6 (6)^†^	− 2 (− 5 to 2)	− 2 (− 6 to 2)	− 1 (− 5 to 2)
Non-injured leg	0 (5)	2 (6)	2 (5)	5 (6)^†^	4 (5)^†^	8 (6)^†^	− 3 (− 6 to 1)	− 3 (− 7 to 1)	− 4 (− 8 to − 1)

Mean (SD) of each group, mean (SD) difference within each group, and mean (95% CI) difference between groups

*Exp* experimental group, *Con* control group

^†^Different compared to pre-surgery (*p* < 0.05)

^a^The composite score is expressed as the mean reaching distance, relative to leg length, of the the eight directions

## Discussion

Twenty-six weeks of standard care improved neuromuscular leg functions relative to pre-surgery but cross-education, as an adjuvant to standard care, did not further improve these outcomes after ACL reconstruction. Remarkably, cross-education had a negative effect on the CAR at 12 weeks post-surgery and decreased the limb symmetry index for maximal quadriceps strength at 5 and 12 weeks post-surgery.

### Primary outcome

Isometric quadriceps MVCs of the reconstructed and non-injured leg did not differ between the experimental and control group but limb symmetry was decreased 9–10% more for the experimental than control group at 5 and 12 weeks after surgery. The present study was designed to examine the long-term effects of cross-education in recreational athletes, whereas a previous study focused on the short-term effects in highly trained soldiers (Papandreou et al. 2013). Unlike in the present study, they found that cross-education in addition to standard care resulted in a quadriceps strength-sparing effect and reduced asymmetry at 8 weeks post-surgery (Papandreou et al. 2013). This strength-sparing effect was evident when cross-education training was performed three and five times per week but no dose–response relationship was observed (Papandreou et al. 2013). Our patients were recreational athletes who trained on average two times per week. This lower cross-education training dose did not attenuate strength loss compared to the standard care group, suggesting that cross-education training should be performed at least more than two times a week to induce a strength-sparing effect. However, how and if at all in the study of (Papandreou et al. 2013) it was cross-education that improved quadriceps strength in the reconstructed leg is unclear, because, unlike previous cross-education studies (Carroll et al. 2006; Manca et al. 2017), cross-education actually occurred in the absence of a training effect in the trained leg (Papandreou et al. 2013). A cross-education effect in the trained leg was also absent in the present study and suggests that the reduction in limb symmetry for the experimental group was not related to the cross-education intervention.

The rate of change in quadriceps MVCs, relative to pre-surgery, showed a different pattern in the two legs. The reconstructed leg revealed 35% and 12% deficit at, respectively, 5 and 12 weeks post-surgery and 11% improvement at 26 weeks post-surgery. The non-injured leg showed a gradual increase in quadriceps strength up to 14% at 26 weeks post-surgery. A training program that solely focused on improving concentric quadriceps strength showed a 13% improvement after 12 weeks of training (Hortobagyi et al. 1996). The non-injured leg of both groups in the present study showed a 12% gain in quadriceps strength at 12 weeks of rehabilitation which suggests that the standard care program effectively increased quadriceps strength and that the contribution of the two extra cross-education exercises was too small to induce extra strength gains in the non-injured leg. Only a few studies report the time course of quadriceps MVCs after ACL reconstruction (Chung et al. 2015; Harput et al. 2015; Lee et al. 2015), so our data elucidate the longitudinal strength development of the reconstructed and non-injured leg. An isometric quadriceps torque of at least 3.0 Nm/kg is related to good patient-reported outcome after ACL reconstruction (Pietrosimone et al. 2016). The patients in the present study scored 3.4 Nm/kg, indicating that quadriceps strength was recovered well 26 weeks after ACL reconstruction.

### Voluntary quadriceps activation

In addition to standard care, cross-education decreased the reconstructed leg’s CAR, suggesting that cross-education cannot attenuate activation failure, a possible cause of quadriceps weakness (Palmieri-Smith et al. 2008). However, the effect of cross-education on the CAR was only observed 12 weeks post-surgery, which makes it of small clinical relevance. At 5 and 12 weeks post-surgery, the twitch interpolation technique provided evidence that the voluntary drive to the quadriceps was reduced and the size of the potentiated twitch force indicated quadriceps weakness. These activation deficits were only observed in the reconstructed leg.

A cross-education intervention in healthy subjects showed a trend towards an increased CAR in the contralateral untrained quadriceps (Lepley and Palmieri-Smith 2014) but ACL reconstructed patients did not show such effect. Instead, cross-education reduced the CAR by 6% at 12 weeks post-surgery compared to standard care. It could be that the neurophysiological alterations following ACL reconstruction (Needle et al. 2017; Nyland et al. 2017) reduce the responsiveness to cross-education training. Especially, because maladaptive changes in somatosensory areas (Baumeister et al. 2008; Grooms et al. 2015b) and motor areas (Grooms et al. 2015b; Lepley et al. 2015; Pietrosimone et al. 2015) are observed after ACL reconstruction and these areas also play a key role in cross-education (Zult et al. 2014). These changes in sensorimotor areas might reduce the sensitivity to sensory cues and motor stimuli, which would diminish the effects of a motor intervention and could explain why cross-education training did not increase the voluntary drive to the quadriceps of the reconstructed and non-injured leg.

The CAR and not the twitch interpolation technique has been widely used in ACL studies (Hart et al. 2010; Lepley et al. 2015). A systematic review showed that the CAR is on average 84% and 89% for the reconstructed and non-injured leg, respectively (Hart et al. 2010). The CAR of our ACL patients at 26 weeks post-surgery was higher and even above the 95%-threshold, a marker of healthy quadriceps function (Hart et al. 2010).

### Quadriceps force control

Our data support the idea that resistance training can improve force control of the quadriceps (Hortobagyi et al. 2001). Force accuracy and variability improved in the reconstructed and non-injured leg by 12 (17–56%) and 26 (22–34%) weeks post-surgery relative to pre-surgery. In addition, force control at week 12 is already better than reported in healthy controls (Zult et al. 2017). Previous studies reported impaired force accuracy (Perraton et al. 2017) and variability (Bryant et al. 2009) in the reconstructed leg 16–18 months post-surgery and that less-accurate force output was associated with worse self-reported knee function and hop test performances (Perraton et al. 2017). Inadequate force control increases knee joint loadings, which, over time, could initiate or accelerate knee osteoarthritis (Tsai et al. 2012). Future research should investigate if ACL reconstructed patients with good force control are indeed less vulnerable to develop knee osteoarthritis.

### Knee joint proprioception

As expected, cross-education training did not improve knee joint proprioception compared to the control group. Whether knee joint proprioception is affected at all after ACL reconstruction is debatable as a recent meta-analysis found no evidence that proprioceptive function was impaired after ACL surgery compared to healthy controls (Nakamae et al. 2017). Knee joint proprioception in the present study was not different before vs. after ACL reconstruction, supporting the idea that ACL surgery does not affect joint position sense.

### Single-leg balance

Rehabilitation did not improve static balance but dynamic balance, measured by the star-excursion balance test and showed an improvement of ~ 7% after 26 weeks of standard care relative to pre-surgery. This improvement is likely the result of the balance training that patients performed as part of their standard rehabilitation program. However, both legs still showed a ~ 6% (0.42 SDs) deficit at 26 weeks post-surgery relative to healthy controls (Zult et al. 2017), confirming previous findings (Clagg et al. 2015). In addition, both legs had a star-excursion balance test composite score of below 94% leg length, which means that our ACL patients were at increased risk to sustain a lower extremity injury (Plisky et al. 2006). Rehabilitation programs should focus on improving dynamic balance before ACL reconstructed patients return to full sport participation.

### Study limitations

The random group allocation resulted in a skewed sex distribution between groups by eight more females in the control vs. experimental group. Compared to males, females have reported worse knee function after ACL reconstruction (Ageberg et al. 2010), were less likely to return to pre-injury sports level (Brophy et al. 2012), and were at increased risk to sustain a second ACL injury (Brophy et al. 2012; Paterno et al. 2012) especially to the contralateral leg (Paterno et al. 2012). The cause of these sex differences is unclear (Di Stasi et al. 2015) but might have biased the results of the control group.

Another factor that could have influenced our results is that a different contraction mode was used in the cross-education training (i.e., concentric) than during strength testing (i.e., isometric). Concentric strength training of the quadriceps in healthy subjects resulted in 30% and 22% cross-education when tested in, respectively, the concentric and isometric mode (Hortobagyi et al. 1997). We have chosen for the isometric testing mode, because ACL patients would be able to perform this test with the reconstructed leg at 5 weeks post-surgery (Harput et al. 2015). In addition, recent evidence shows that training of the non-immobilized wrist flexors in one mode results in strength preservation across all contraction modes in the non-trained wrist flexors following 4 weeks of immobilization (Andrushko et al. 2017). Altogether, it is unlikely that we would have found a cross-education effect for strength if we had tested the quadriceps in the concentric mode.

Neurophysiological adaptations, except voluntary quadriceps activation, were not examined in the present study. Imaging, electroencephalographic, and transcranial magnetic stimulation studies will shed light on the cortical and corticospinal responses of therapeutic interventions after ACL reconstruction. Such studies are needed as maladaptation in sensorimotor areas appear to be associated with functional deficits after ACL reconstruction (Needle et al. 2017; Nyland et al. 2017). Understanding whether therapeutic interventions are able to enhance motor planning, sensory processing, and visual motor control will improve rehabilitation outcomes after ACL reconstruction and will decrease the ACL reinjury risk (Grooms et al. 2015a).

## Conclusion

Twenty-six weeks of standard care, specifically targeting the reconstructed leg, recover neuromuscular function but cross-education in addition to standard care did not further improve the recovery process after ACL surgery. Maladaptation in sensorimotor areas following ACL reconstruction might reduce the sensitivity to sensory cues and motor stimuli, which will decrease the responsiveness to motor interventions like cross-education. Perhaps when the nervous system is intact, e.g., in the immobilization phase after a wrist fracture, a cross-education intervention will have greater efficacy.

## Abbreviations

ACL: Anterior cruciate ligament
CAR: Central activation ratio
MVC: Maximal voluntary contraction
SD: Standard deviation
